# Investigation of genetic markers for intramuscular fat in the hybrid Wagyu cattle with bulked segregant analysis

**DOI:** 10.1038/s41598-021-91101-w

**Published:** 2021-06-01

**Authors:** Yun Zhu, Liyun Han, Peng Li, Xiaolong Kang, Xingang Dan, Yun Ma, Yuangang Shi

**Affiliations:** 1 School of Agriculture, Ningxia University, Helan Mountain West Road 489, Hui Autonomous Region, Yinchuan, 750021 Ningxia China; 2 Pingjibao Dairy Farm, Ningxia Agricultural Reclamation Helanshan Dairy Co. Ltd, Xixia District, Hui Autonomous Region, Yinchuan, 750021 Ningxia China

**Keywords:** Haplotypes, Genetic hybridization, Genetic linkage study, Genome, Genotype, Genetics, Genetic association study, Genome-wide association studies

## Abstract

ulked Segregant Analysis (BSA) is a rapid strategy for identifying genetic markers in specific regions of the phenotypical population and it has been widely used for QTLs mapping in smaller mixed F2 and F3 populations. We applied a modified BSA method to assessed genome-wide homozygous and heterozygous linkage patterns in the Chinese Wagyu Beef Cattle F2/F3 mixed population. Two overlapped regions from F2 and F3 populations on autosomes were found with high-density heterozygote alleles between high and low intramuscular fat groups. Regions from 24.8 M ~ 29.6 M of chromosome 23 were identified as most significantly correlated to the intramuscular fat in our samples. We also identified other 4 potential loci on chromosomes 5, 9, 15, and 21 correlated with Intramuscular fat. This study provided a novel low-cost method for QTLs mapping and identify molecular markers of phenotypical changes in a small mixed population.

## Introduction

Bovine are one of the most important economic animals in the world. It serves the community by supplying milk, meat, leather, and nutrition to the human body. After years of development, Bovine has led to different breeds with a variety of phenotypes in the different geographical regions. Natural selection has led to an increase in cattle diversity. At the same time, artificial selection has greatly been implemented to maximize the economic benefits of cattle. Many breeds have been successfully achieved to accommodate the demands of specific traits. This study introduced the frozen sperm of Japanese Wagyu cattle hybridized with Qinchuan and Luxi cattle in order to improve the potential of the quality of meat.

Intramuscular fat (IMF) of meat is distributed in the form of white marbling, commonly known as marbling, and is one of the important indicators for meat quality. The richer the marbling, the higher the IMF content of meat and the better meat quality. IMF deposition and distribution are regulated by heredity, age and epigenetics. The word “marbling” is defined by the amount and distribution of visible IMF that accumulated within the muscle and muscle fiber bundles. It is an important economic trait in all cattle and stands out particularly in the Japanese cattle^1^. IMF content has become one of the most important indexes for meat quality evaluation^2^. It affects the meat tenderness, hydraulic power, shearing force, flavor, and juiciness^3^. To impove the IMF in Chinese beef cattle and understand the regulation mechanism, the researches on IMF have become a hot topic in recent years.

Qinchuan and Luxi cattle are the local breeds in China. They are both steer, less intramuscular fat deposition However, in comparison with Japanese wagyu cattle, Qinchuan and Luxi cattle have smaller transection area of the longissimus dorsi muscle and lower IMF content. Nevertheless, there is one disadvantage of Qinchuan cattle, IMF deposits require longer feefing times (40 month). Japanese wagyu cattle are now being brought in to cross-breed with local Chinese cattle in the hope of improving the quality of meat. A significant improvement was found in meat quality in filial generation. The hypothesis that the majority of IMF content in these offspring should be located in between Qinchuan and purebred Wagyu cattle, and some cattle could exhibit the extreme phenotypes on both ends. Due to the limitation of GWAS, it was difficult to locate the genes associated with IMF. Therefore, the potential SNPs in Chinese hybrid waygu cattle were analyzed by BSA.

Bulked Segregant Analysis (BSA) is a method of rapid gene mapping for target traits inindividuals with extreme phenotypes in hybrid populations. The whole genome was sequenced on the two sample pools with extreme traits and the DNA fragments between the two mixed pools were detected to be the candidate regions. In recent years, BSA has been widely adopted for DNA and RNA analysis and has been successful used in genemapping and single nucleotide polymorphism(SNP) mapping^4^. BSA has a significant contribution in understanding animal genetics^5^, genomics, crop breeding, and improve productivity^6–8^. BSA analysis with high-throughput sequencing is an attractive tool to most scholars. Incomplete dominance, also known as semi-dominance, is the recessive genes that can express in the heterozygotes' progeny. Coat color has been researched as semi-dominant inheritance in cattle^9^.

In this study, a modified BSA-based method was applied for IMF QTLs mapping. Both F2 and F3 cross-population of Luxi, Qinchuan and Wagyu were used for bulk allele frequency difference analysis. Several QTLs were found correlated to extreme high and low IMF. 5 Mb of chromosome 23 was identified as the most significant QTLs of IMF in two paired bulk (both F2 and F3) sequencing. There were six highly expressed fat genes and two highly expressed muscle genes in this region.

## Methods

### Population samples

In total, 186 samples of Chinese wagyu beef cattle (Both F2 and F3 cross-population of Luxi, Qinchuan and Wagyu) were taken from three different farms in China (Shandong Kaiyuan Co. Ltd., Shandong Province, China; Ningxia Yijiayi Farming and Animal Husbandry Co. Ltd., Ningxia Hui Autonomous Region, China; Ningxia Xuanheyuan Agriculture and Animal Husbandry Co. Ltd., Ningxia Hui Autonomous Region, China). The background information of samples is shown in Table 1.

**Table 1:** The background information of samples.

Dam	Male	Number	Name of the cattle farmer
Qinchuan	Wagyu	113	Ningxia Yijiayi Farming and Animal Husbandry Co. Ltd., Ningxia Hui Autonomous Region, China
Qinchuan × Wagyu	Wagyu	40	Ningxia Xuanheyuan Agriculture and Animal Husbandry Co. Ltd., Ningxia Hui Autonomous Region, China
Qinchuan × Wagyu × Wagyu	Wagyu	33	Shandong YuanLong Co. Ltd., Shandong Province, China

**Figure 1:**
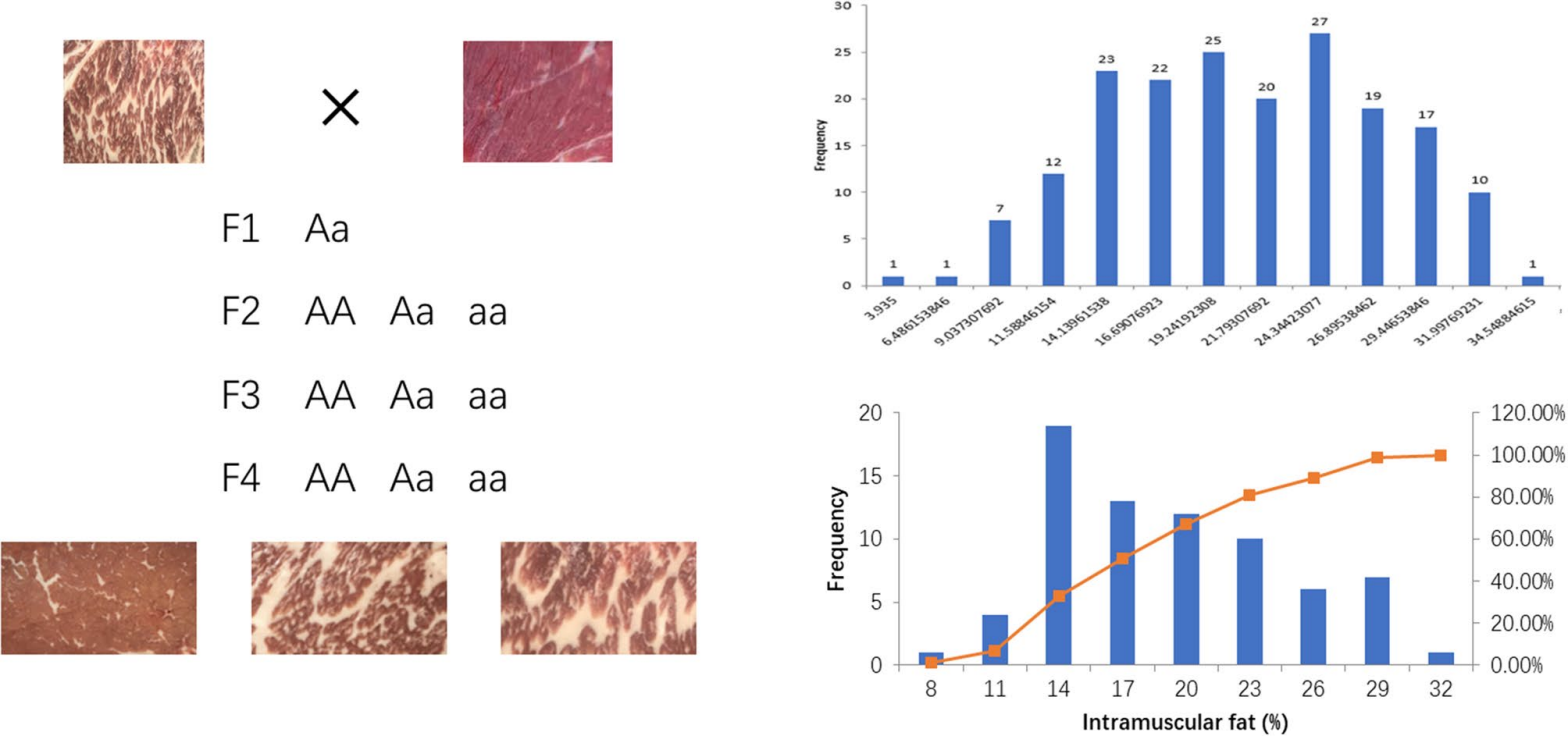
Population distribution of samples (left) and each sample frequency distribution of intramuscular fat (right).

Cattles were hybridized for four generations according to Fig. 1. The castration procedure was performed at 5 months of age and slaughtered at 28 months. Blood and longissimus dorsi muscle tissues were collected after slaughtered immediately.

Due to the limitation of cattle farms hybridization program, it is very difficult to take experimental samples from one single farm, therefore three farms were selected. The source of wagyu beef sperm came from Beijing Dairy Cow Center (BDCC). The animal use protocol listed below has been reviewed and approved by the Animal Ethical and Welfare Committee (AEWC). NO. IACUC-NXU1014, and all cattle were electric shocked before slaughter, Electric shocks can make cattle painless when they are slaughtered, this test conform to the requirements of American Veterinary Medical Association (AVMA) Guidelines. Samples collected in this experiment have been given permission from respective farms in China from where of Chinese Wagyu Beef Cattle. I've provided three statements to confirm that permission was obtained from respective farms in China from where the of Chinese wagyu beef cattle. Three statements are uploaded in the Supplementary Materials and Related Files section.

### Phenotypes determination

Phenotype determination was obtained after acid excretion and segmentation. In short, 100 g longissimus forsi were collected, and then intramuscular fat (IMF) content was determined according to the national fat content determination standard. The intramuscular fat content was determined following the guideline of Chinese national standard GB/T 9695.7–2008 in the booklet “Determination of Total Fat in Meat and Meat Products”. The crude fat content was determined by Soxhlet method.

### DNA sequencing

A total of 2 µg genomic DNA (35 ng/ml) was fragmented into 300 bp using the Bioruptor UCD-200 sonicator (Diagenode, Denville, NJ) for each sample. Library preparation was constructed using the Kapa Hyper DNA library prep kit for the Illumina platform. Fragmented DNA was end-repaired with an end-repair enzyme and a deoxyadenosine was added to the 3’ ends of the fragments. Kapa barcoded DNA and Kapa indexed adapters were ligated to the sample libraries. The adapter-ligated libraries were selected for an average insert size of 300 bp using next-generation sequencing cleanup and size selection kit (NucleoMag, Macherey–Nagel, Duren, Germany) according to the manufacturer’s protocols. The quality of libraries was assessed using the Bioanalyzer 4200 (Agilent Technologies, Santa Clara, CA). The libraries were then quantified by qRT-PCR and then sequenced were performed by Illumina Nova-seq platform to generate 150-bp paired-end reads.

### Sequencing genotypes calling

**Figure 2:**
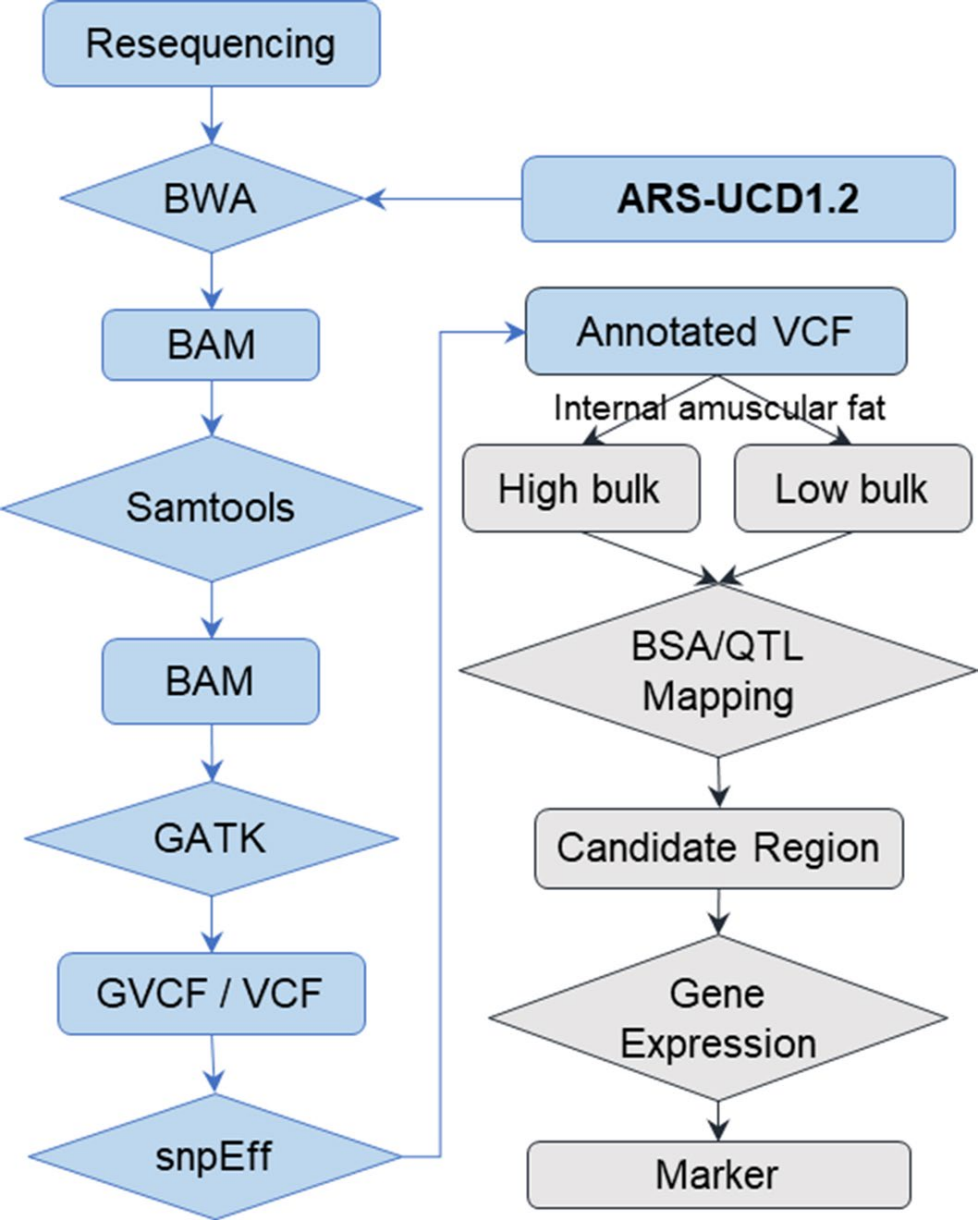
Workflow of variants calling (blue boxes) and QTL mapping (grey boxes).

Raw sequence reads were processed using Fastp(v0.12.4)^10^ to remove low-quality reads and adapters. BWA (v0.7.16)^11^ mem (-M -a) module was applied to align the high-quality reads to ARS-UCD1.2 cattle genome with default parameters. SAMtools (v1.9)^12^ were used to sort BAM files, remove read of PCR duplications and for sample alignment statistics. “BaseRecalibrator” and “ApplyBQSR” module of GATK (v4.0.10.1) were used for base quality score recalibration. “HaplotypeCaller” module with minimum-mapping-quality 30 for each sample GVCF. After that, raw cohort VCF was worked out with modules “CombineGVCFs” and “GenotypeGVCFs”. Bcftools^13^ (v1.9) was performed as variants quality filtering with "QUAL >  = 100". To annotate variants, ensemble gene annotations base on ARS-UCD1.2 were used for the database which was created by snpEff (v4.3T)^14^. The brief workflow of variants calling and annotation was shown as blue boxes in Fig. 2.

### BSA-based allele frequency analysis

The allele frequency of each population was extracted from the VCF file by bcftools query module. DFScore defined as the variance of allele frequency in a sliding window. When the allele frequency in a window obeys the frequency expectation of two bulk samples or the variance in each bulk sample close to or equal to zero, we treat it as the best window. The benefit of this calculation is that we can more accurately screen segments that meet genetic expectations. For instance, assumed the data is from an F2 population, the allele frequency expectation of dominant bulk should be 1/3 or 2/3 and the allele frequency expectation of recessiveness bulk should be 0 or 1. Similarly, if the data were generated with homozygous samples in the two extreme phenotype bulks, the allele frequency expectation of the two bulks will be either 0 or 1. The benefit of this calculation is that we can more accurately screen segments that meet genetic expectations. SNP density was plot by R package CMPlot (https://github.com/YinLiLin/CMplot) with 1 M bp as windows size. Variance analysis of allele’s frequency was flowed by varBScore^15^, windows size was set as 10 SNP and step size was 5 SNP. Power AFD was modified by ED algorithm and inherited the power 4 to reduce the background noise^7^.

The $PowerAFD$ can be calculated as follows:

$$PowerAFD=|FREQ\,of\,High\,IMF\,Group-FREQ\,of\,Low\,IMF\,Group|^4$$

### Selection of relevant SNP windows and putative candidate genes identification

Calculate the SNP density with the allele frequency difference between the two paired-bulks was setting up with the filter premaster as AF =  < 0.45 or AF >  = 0.55. 1 Mb was set as slide window length on the genome to evaluate the SNP density with a difference in frequency of each allele.

### Gene expression analysis

Gene expression conversion and general expression summary in annotated tissues was obtained on the cattle gene expression database from Bgee (https://www.bgee.org/). In total, 112 muscle and fat tissues of cattle RNA-seq datasets were downloaded from NCBI and processed to the FASTQ format using the NCBI sratoolkit (version 2.9.6). Then low-quality reads and adapters were removed using the Fastq program (version 0.12.4,)^10^. Kallisto (version 0.45.0)^16^ was used to quantized gene expression. Ensembl genome bos_taurus.ARS-UCD1.2 and gene annotation version 100 were used as references gene. Gene expression heatmap was generated by R package “pheatmap” (version 1.0.12) with gene TPM.

## Results

### SNP

Extreme high and low IMF samples (16 samples from F1, 14 samples from F2, and 13 samples from F3) were sequenced with an average of 10X coverage with reference genome per sample. Total 1075G raw sequencing data were obtained. Cohort variants calling was performed with GATK4 best-practice pipelines. SNP and InDel were worked out with reference bos_taurus.ARS-UCD1.2 for all samples. To apply BSA-based QTL mapping, samples from the same generation population were extracted into a sub VCF. Three paired-bulk datasets (F1 high IMF bulk vs F1 low IMF bulk; F2 high IMF bulk vs F2 low IMF bulk; F3 high IMF bulk vs F3 low IMF bulk) were used for allele frequency analysis in the next step.

### Allele frequency difference (AFD) analysis

Calculate the SNP density with the allele frequency difference between the two paired-bulks was setting up with the filter premaster as AF =  < 0.45 or AF >  = 0.55. 1 Mb was set as slide window length on the genome to evaluate the SNP density with a difference in frequency of each allele. The highest density AFD SNP was 43/Mb of F2 bulks and 55/Mb of F3 bulks. There were 4 chromosomes in the F2 population and 5 chromosomes in F3 had AFD-SNP density greater than 30/Mb. Among them, chromosome 7 appeared in both groups, however, the specific segments were inconsistent. Chromosome 23 also appeared in the two populations, and the segments were very close to each other. AFD-SNP appeared in the same segment on chromosome 19 in F2 and F3. Also, F3 had a high density of SNPs in the segment of 322 M in chromosome 27 (Table 2).

**Table 2:** High density of SNP between two groups (> 30 SNP/Mb).

Group	Chrome	Start	End
F2	7	95,600,000	97,000,000
F2	15	50,600,000	52,400,000
F2	19	55,600,000	57,400,000
F2	23	25,200,000	29,600,000
F3	4	105,200,000	106,200,000
F3	7	5,000,000	6,600,000
F3	19	55,200,000	56,600,000
F3	23	24,800,000	29,600,000
F3	27	32,200,000	34,000,000

**Figure 3:**
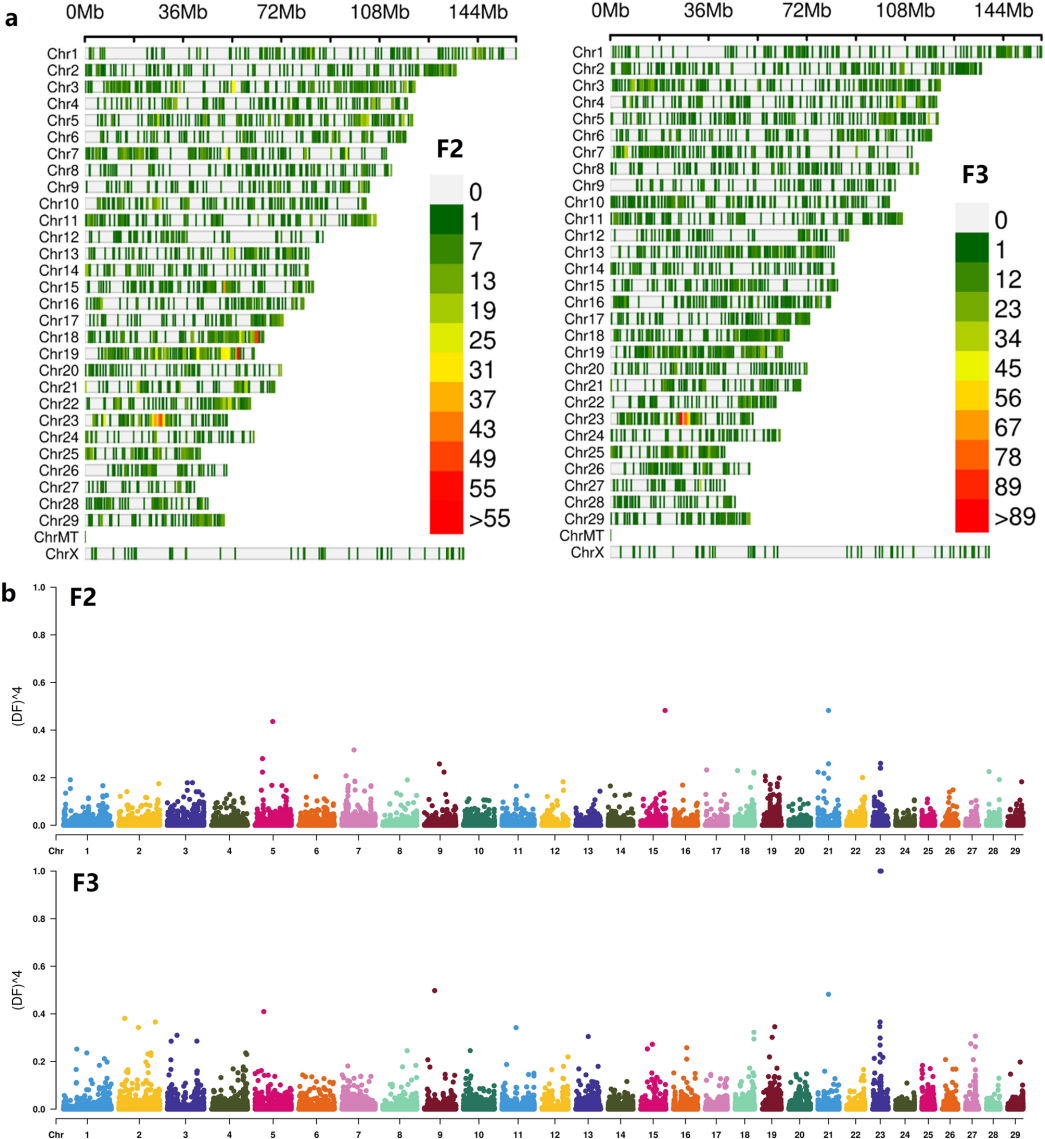
(**a**) SNP density plot (Filter SNP with allelic frequency difference >  = 0.3 in two groups). Figures from left to right were the population F2 and F3; (**b**) QTL-mapping of allele frequency difference of F2, and F3 population with the degree of freedom.

Further analysis of the density distribution of SNPs with AFD >  = 0.3, it can be found from Fig. 3 that high-density SNPs appeared in the middle of chromosome 23 in both populations. Among them, there is significant enrichment on chromosomes 18 and 19 in the F2 population. Look at the F2 group as a whole. In the F2 population, there are 3 segments with SNP density greater than 50/M. In the F3 population, there is only one very significant SNP-enriched segment, but the number of SNPs exceeds 90 (Fig. 3a).

**Figure 4:**
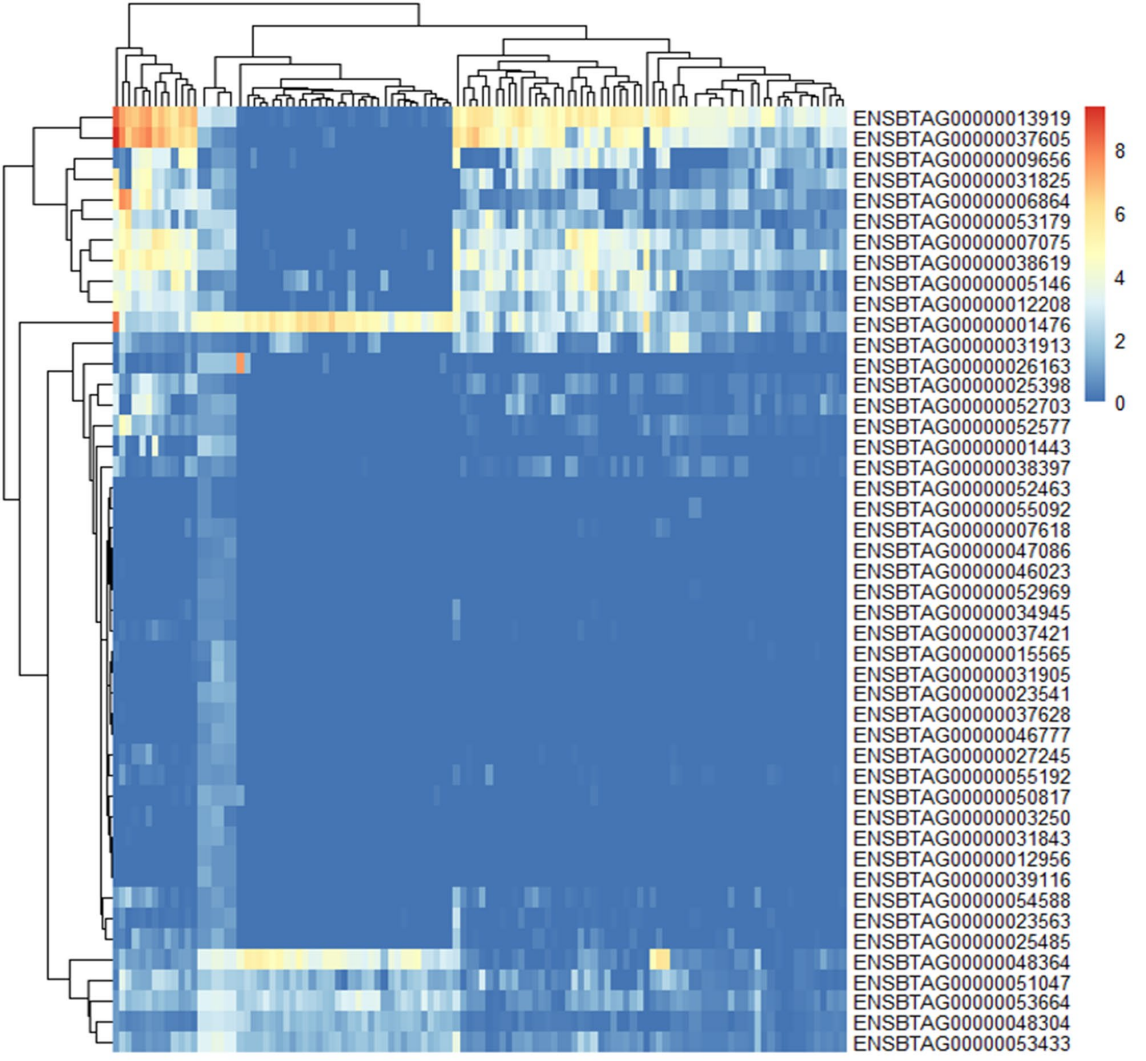
Expression pattern of 46 gene in chromosome 23: 24.8 M ~ 29.6 M.

In order to observe the sites and segments of genotype differences more significantly, power4 (AFD) analysis based on SNP sites with AFD >  = 0.3 to amplify the ratio of site allele frequency differences. After visualazed the power4(AFD) with CMPlot (Fig. 4), we found that there were several low significantly score of power4(AFD) (> = 0.3) locus on chromsome 5, 15, 21, 23 in F2 bulk. In F3 bulk, locus on chromosome 9 and 23 become more significant difference (Fig. 3b, Table 3). In total 16 SNP of F3 were found powe4(AFD) >  = 3. Two alleles on chromosome 21 are neared and two locus of chromosome 23 were closed.

**Table 3:** Loci and statistical power (DF >  = 0.3).

Population	Chrome	Pos	Power4(DF)
F2	5	58,695,696	0.4358063
	7	40,433,553	0.3164062
	15	81,148,712	0.4822531
	21	34,817,429	0.4822531
F3	2	19,661,262	0.3811172
	2	65,195,647	0.3426863
	2	121,333,197	0.3659503
	3	31,979,076	0.3099656
	5	29,006,626	0.4096
	9	34,246,206	0.4978714
	11	46,714,219	0.342135
	13	42,035,405	0.3048815
	18	63,302,729	0.3226693
	19	32,997,278	0.3016062
	19	41,594,141	0.3458889
	21	34,817,458	0.4822531
	21	34,817,459	0.4822531
	23	24,479,417	0.3465916
	23	25,583,794	0.3659503
	27	33,107,854	0.3064806

**Figure 5:**
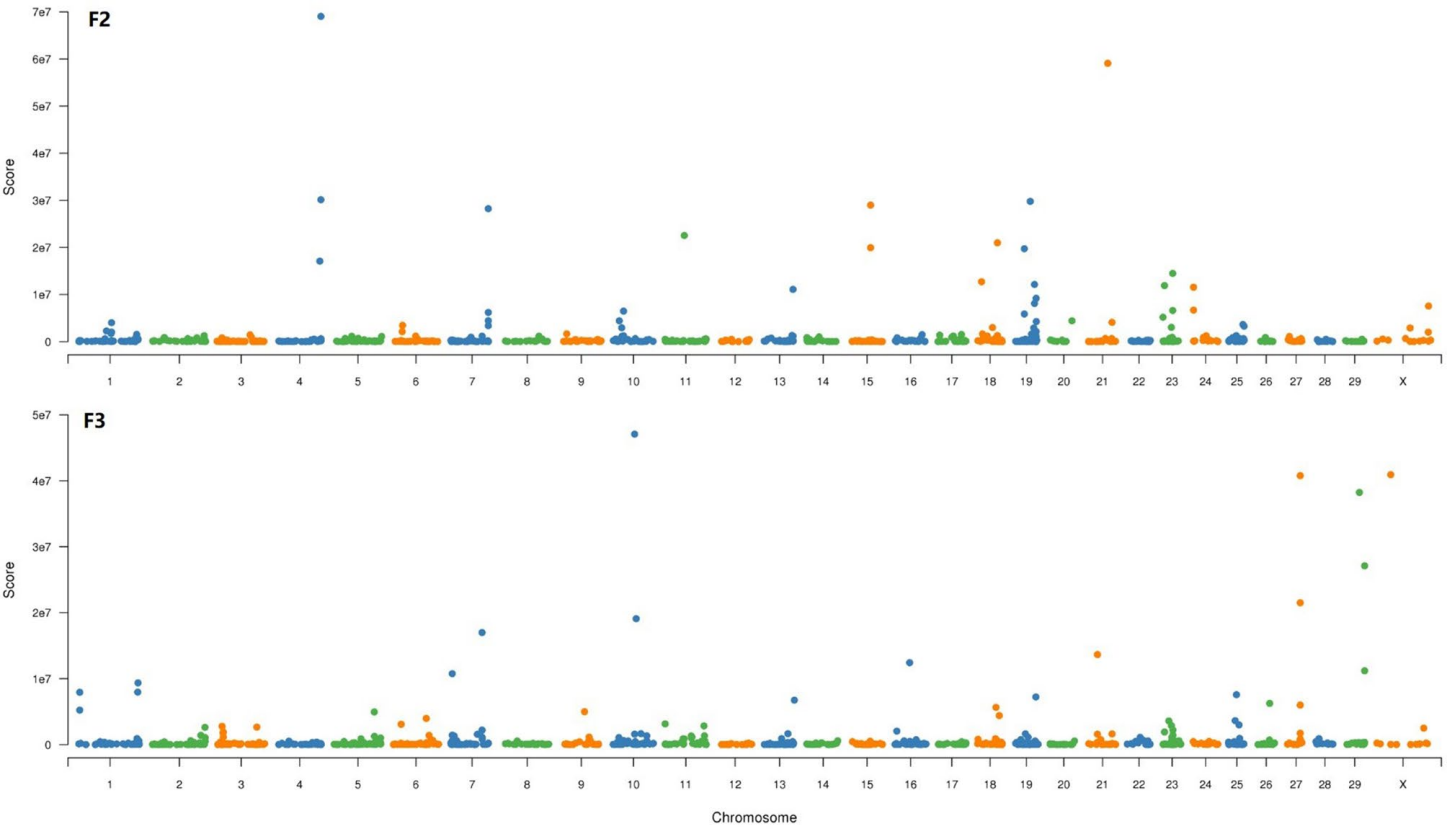
Variance analysis of allele frequency in 10 SNP slide windows.

During the haplotypes analysis, we found more segments were associated with target traits in the mixed pools of the two groups. However, only a few segments were overlapped in two groups. To rationale our finding, it may be related to small sample cohorts and some background noise. Nevertheless, our finding showed the chromosomes 7, 10, 13, 19, 21, and 23 are relatively important to the IMF (Fig. 5). It was confirmatory to our finding in Fig. 3a (chrome 19, 21, 23, etc.).

It has been shown that most of the F1 should inherit 50% of genes from each parent. The genotype of indel from the hybrid F1 is biased as homozygous, and such genotype cannot be used to calculate AFD in the two extremes traits.

### Target region gene expression

As chromosome 23 consistently appeared in two generations, we investigated this target region with further analysis. There were 212 genes on region from 24.8 M ~ 29.6Mof chromosome 23. 177 genes were expression in muscle tissues or fat tissue (at least one of 112 sampleshowed log2(TPM) > 1). 46 genes were identified with AFD SNP between two group. Six genes (ENSBTAG00000001476, ENSBTAG0000048364, ENSBTAG0000051047, ENSBTAG0000053664, ENSBTAG0000048304, ENSBTAG0000053433) were found high expression in fat and two genes (ENSBTAG0000013919 and ENSBTAG0000037605) were found highly expressed in muscle (max log2(TMP) > 8) (Fig. 4). 35 of 112 genes in candidate region not significant expressed in muscle and fat tissues (max log2(TPM) < 1).

## Discussion

BSA has been used to identify molecular markers in a wide range of organisms.Many methods and pipelines have been developed in model plants Arabidopsis and rice^17^. In the classical BSA analysis, researchers usually first mix individuals with the same traits in equal amounts of DNA for next-generation sequencing and allele frequency analysis. By mixing the samples, it can minimize the costs of next-generation sequencing. On the contrary, we sequenced all individuals with extreme traits. The overall data of the same phenotype is later mixed to ensure the consistency and coverage of sequencing. Our data suggested that sequencing individual samples may be a better option to ensure data consistency and sequencing depth.

In particular, marbling is the primary factor for determining meat quality and price. The IMF content is positively correlated with marbling score (MS). Some of which have been previously reported MS was also localized on Chr. 23^18^. In addition, there has been a population-related study of IMF in Wagyu cattle. Thyroglobulin (TG) gene is located on the quantitative trait loci affecting IMF content and encodes key factors in the metabolic pathway^19^. Another research for body height, two significant Runs of homozygosity(ROHs) were found at BTA23 and BTA7, but fat coverage at BTA28^20^. The possible reason is that the population combinations are completely different and different genetic background generate different regulatory mechanism. Quantitative traits, such as IMF content, are influenced by interaction between genotype and environment, nutrition also has an effect on the population. From the mothed point of view, Traditional SNP GWAS provides limited genetic information. To increase the sensitivity of region/segments with SNP linkage in our F2/F3 populations, we used the haplotypes which contained 10 adjacent SNPs as a window, and calculated individual SNP frequency based on the sliding window. In general, chromosome recombination and exchange occur randomly and independently and can be very different in each individual. The sliding windows that were significant associated with the population may be related to our target traits.

In the classical BSA analysis, researchers usually first mix individuals with the same traits in equal amounts of DNA for next-generation sequencing and allele frequency analysis^7^^,^^16^. By mixing the samples, it can minimize the costs of next-generation sequencing. On the contrary, we sequenced all individuals with extreme traits. The overall data of the same phenotype is later mixed to ensure the consistency and coverage of sequencing. Our data suggested that sequencing individual samples may be a better option to ensure data consistency and sequencing depth.

## Conclusions

This study provided a BSA-based QTL mapping method to analyze the genetic marker of IMF in the different generation (F2/F3) cross populations between Luxi, Qinchuan and Wagyu. Following Mendelian's law of inheritance, the allele frequencies of the hybrid populations were also being investigated, and those AFD may be associated with the phenotypic trait of IMF which had a potentially high economical value of cattle. Multiple potential QTL loci were found. For better association analysis and marker validation, it is recommended that more sequence of extreme traits is needed to obtain better resolution.

## Supplementary Information

Supplementary Information 1.

Supplementary Information 2.

Supplementary Information 3.

Supplementary Information 4.

Supplementary Information 5.
