# Supplementary material for: Investigation of genetic markers for intramuscular fat in the hybrid Wagyu cattle with bulked segregant analysis
Source: Sci Rep. 2021 Jun 1;11:11530. doi: 10.1038/s41598-021-91101-w (PMC8169923; doi:10.1038/s41598-021-91101-w)
Supplement: Supplementary file 1 — Supplementary Information 1. [file 41598_2021_91101_MOESM1_ESM.pdf]

# STATEMENT

I agreed to allow Yun Zhu from Ningxia University to collect samples of hybrid cattle of Qinchuan  and Wagyu cattle in our farm.

Ningxia Yijiayi Farming and Animal
Husbandry Co. Ltd.,
Ningxia Hui Autonomous Region，
China
