# Supplementary material for: Investigation of genetic markers for intramuscular fat in the hybrid Wagyu cattle with bulked segregant analysis
Source: Sci Rep. 2021 Jun 1;11:11530. doi: 10.1038/s41598-021-91101-w (PMC8169923; doi:10.1038/s41598-021-91101-w)
Supplement: Supplementary file 2 — Supplementary Information 2. [file 41598_2021_91101_MOESM2_ESM.docx]

Dear Karishma Nagpure：

Thanks for your email.

I confirmed that the graphs in figure 1,2,3,4 and 5 were taken by myself (Yun Zhu) from my paper"Investigation of genetic markers for intramuscular fat in the hybrid Wagyu cattle with bulked segregant analysis".

Best regards,

Yun Zhu([real5949@qq.com](NULL))
