# Supplementary figures and images for: Investigation of genetic markers for intramuscular fat in the hybrid Wagyu cattle with bulked segregant analysis
Source: Sci Rep. 2021 Jun 1;11:11530. doi: 10.1038/s41598-021-91101-w (PMC8169923; doi:10.1038/s41598-021-91101-w)

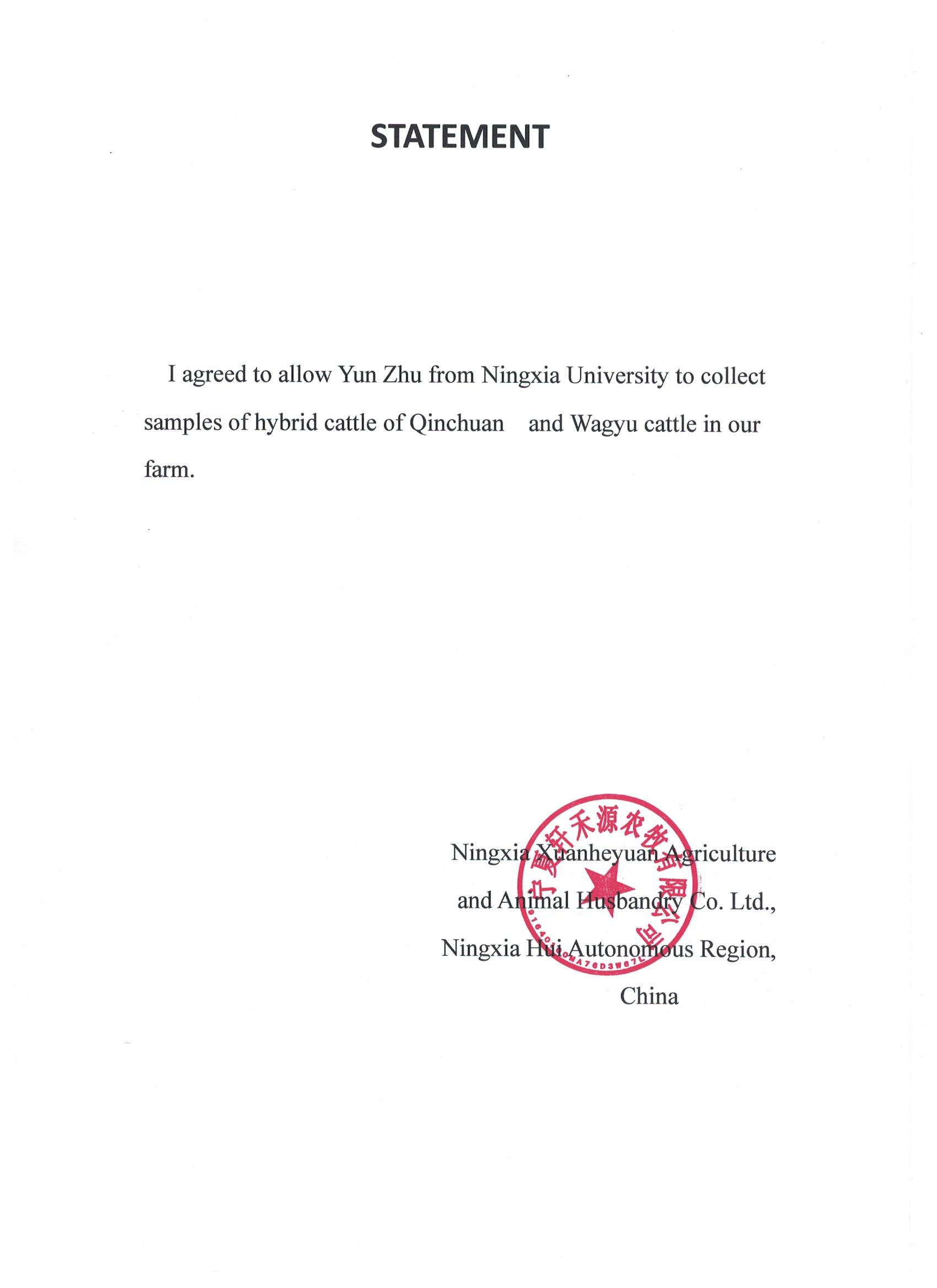

Supplement: Supplementary file 3 — Supplementary Information 3. [file 41598_2021_91101_MOESM3_ESM.docx]
