# Supplementary material for: Investigation of genetic markers for intramuscular fat in the hybrid Wagyu cattle with bulked segregant analysis
Source: Sci Rep. 2021 Jun 1;11:11530. doi: 10.1038/s41598-021-91101-w (PMC8169923; doi:10.1038/s41598-021-91101-w)
Supplement: Supplementary file 4 — Supplementary Information 4. [file 41598_2021_91101_MOESM4_ESM.pdf]

STATEMENT

I agreed to allow Yun Zhu from Ningxia University to collect samples of hybrid cattle of Luxi and Wagyu cattle in our farm.

Shandong YuanLong Co. Ltd.,
Shandong Province,
China;
